# Addressing the batch effect issue for LC/MS metabolomics data in data preprocessing

**DOI:** 10.1038/s41598-020-70850-0

**Published:** 2020-08-17

**Authors:** Qin Liu, Douglas Walker, Karan Uppal, Zihe Liu, Chunyu Ma, ViLinh Tran, Shuzhao Li, Dean P. Jones, Tianwei Yu

**Affiliations:** 1 School of Software Engineering, Tongji University, Shanghai, 201804 China; 2 Department of Environmental Medicine and Public Health, Icahn School of Medicine at Mount Sinai, New York, NY 10029 USA; 3 Department of Medicine, School of Medicine, Emory University, Atlanta, GA 30322 USA; 4 The Jackson Laboratory, Farmington, CT 06032 USA; 5 School of Data Science, The Chinese University of Hong Kong – Shenzhen, Shenzhen, 518172 Guangdong Province China

**Keywords:** Data processing, Scientific data, Computational biology and bioinformatics

## Abstract

With the growth of metabolomics research, more and more studies are conducted on large numbers of samples. Due to technical limitations of the Liquid Chromatography–Mass Spectrometry (LC/MS) platform, samples often need to be processed in multiple batches. Across different batches, we often observe differences in data characteristics. In this work, we specifically focus on data generated in multiple batches on the same LC/MS machinery. Traditional preprocessing methods treat all samples as a single group. Such practice can result in errors in the alignment of peaks, which cannot be corrected by post hoc application of batch effect correction methods. In this work, we developed a new approach that address the batch effect issue in the preprocessing stage, resulting in better peak detection, alignment and quantification. It can be combined with down-stream batch effect correction methods to further correct for between-batch intensity differences. The method is implemented in the existing workflow of the apLCMS platform. Analyzing data with multiple batches, both generated from standardized quality control (QC) plasma samples and from real biological studies, the new method resulted in feature tables with better consistency, as well as better down-stream analysis results. The method can be a useful addition to the tools available for large studies involving multiple batches. The method is available as part of the apLCMS package. Download link and instructions are at https://mypage.cuhk.edu.cn/academics/yutianwei/apLCMS/.

## Introduction

Metabolomics using liquid chromatography-mass spectrometry (LC/MS) is widely used in identifying disease biomarkers, finding drug targets and unravelling complex biological networks. A high-resolution LC/MS profile from a complex biological sample contains thousands of features, and different LC/MS platforms yield profiles of different characteristics. There are a number of computational pipelines that conduct the necessary steps to preprocess LC/MS data, including peak detection, peak quantification, retention time (RT) correction, feature alignment, and weak signal recovery^1–13^. Some methods provide utilities to group features that are potentially derived from the same metabolite^14–17^. Other data servers and packages are available to annotate features to known metabolites based on m/z and RT information^18–21^.

When the sample size is large, it is often necessary for the samples to be processed in batches. Across the batches, even if the data are generated from the same machine, we often observe different data characteristics. Using traditional data preprocessing approaches, we either treat all the samples as a single batch, or preprocess different batch individually, and then seek to merge the feature tables. As we discuss in the following, both of the approaches have some issues.

If we treat all samples as a single batch, the between-batch data characteristic changes will be considered as random noise. More lenient thresholds have to be used in feature alignment and weak signal recovery, in order to tolerate the between-batch differences. This can result in distinct features being artificially merged as a single feature. On the other hand, if a feature has a large drift in RT across batches, it may be artificially split into two features. The issue of misalignment caused by batch effect has been discussed in more detail by Brunius et al.^22^.

An alternative approach is to preprocess each batch individually, followed by alignment of features between the feature tables from separate batches^22^. This approach allows optimal alignment within each batch. However, without between-batch RT correction and weak signal recovery across batches, low intensity features that are initially identified in a subset of batches cannot be accurately quantified in the remaining batches.

Applying batch effect removal methods after preprocessing can alleviate some of the issues. They include methods that use quality control data to adjust for signal drift and inter-batch and intra-batch variations^22–26^, and some methods that use data characteristics without the need for quality control, mainly for between-batch adjustments^27–33^. However, such approaches can only adjust signal intensity. They cannot address issues such as misalignment of features across batches^22^, or the incomplete weak signal recovery from the original data.

To tackle the afore-mentioned problems, we propose a new approach that preprocess the data in a two-stage manner. The method directly uses the batch information to allow optimal within-batch and between-batch alignments. Within each batch, every sample contains a small amount of nonlinear RT drift, which is typically addressed by nonlinear curve fitting^5,11^. Between batches, there may exist systematic RT drift. Both levels need to be adjusted for in the final data matrix. Another major issue is weak signal recovery across batches, as some peaks are too weak to pass the initial detection threshold, but can be later recovered based on the information of their counterparts in other samples. When such information come from other batches, accurate RT correction is critical for the faithful recovery of the weak signal. In our two-stage approach, the RT adjustment is based on cumulative nonlinear curve-fitting, which allows weak signal recovery across batches. Using a dataset from a quality control sample, a yeast cell line dataset, and a dataset generated from healthy human plasma samples, we show the method offers higher consistency in feature quantification for studies involving multiple batches, yielding better results in down-stream analyses.

## Materials and methods

### The overall workflow

**Figure 1:**
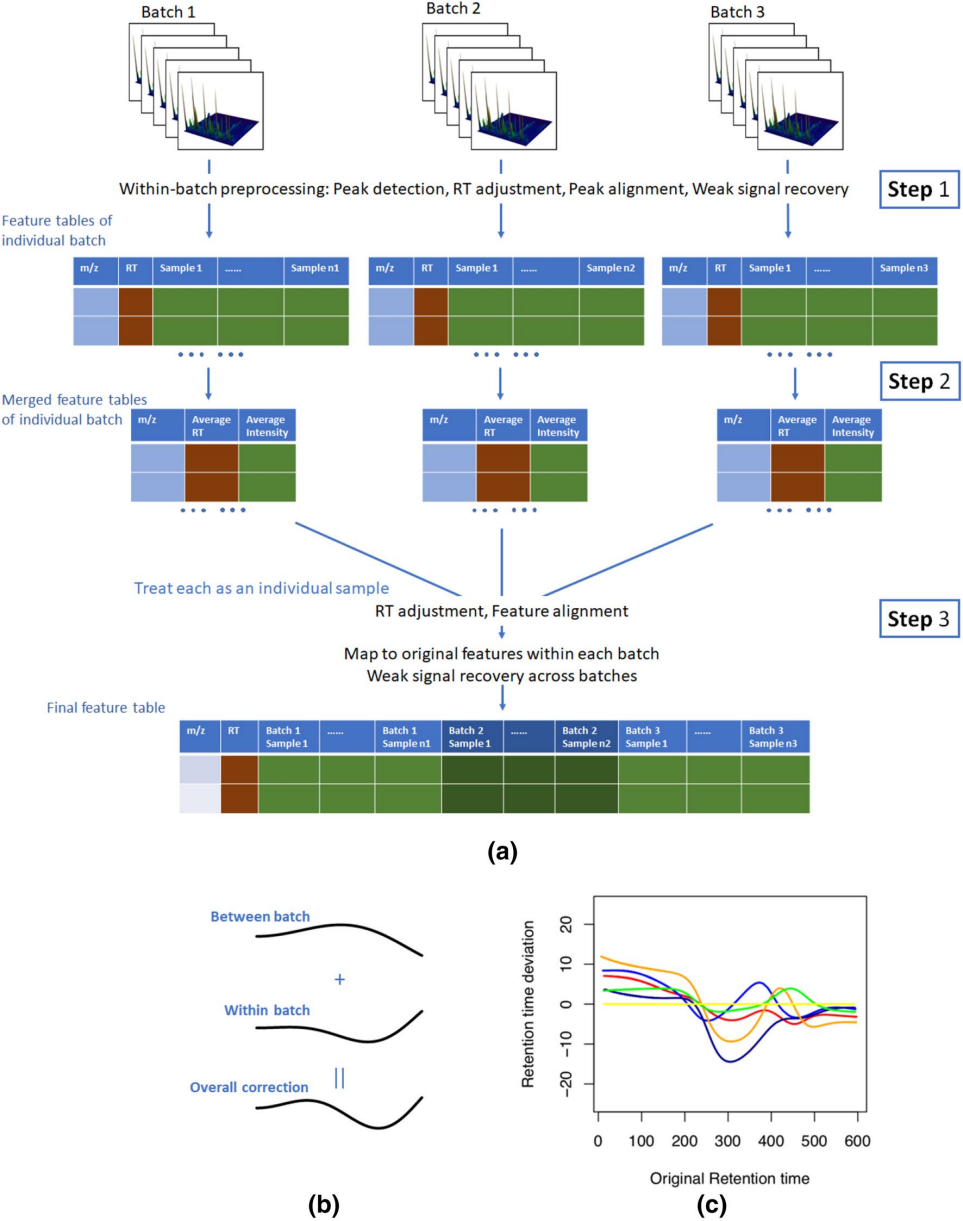
Illustration of the two-stage preprocessing approach. (**a**) The overall workflow. (**b**) Illustration of the calculation of RT shift for individual samples. (**c**) Example between-batch RT shift calculated from a real dataset.

Different from the traditional workflow, the proposed method includes a two-stage procedure (Fig. 1a). In the traditional workflow used by XCMS^5^ and apLCMS^11^, peaks are first identified in the individual profiles based on certain filters, and quantified using certain mathematical peak shape models. Then RT correction is conducted between the profiles, and peaks from different profiles are aligned into features. Then a weak signal recovery step is conducted, in order to capture feature signals that are not strong enough to pass the initial peak detection threshold.

The new approach is divided into two stages. In the first stage (Fig. 1a, step 1), the method processes each batch individually by using the common preprocessing workflow that consists of peak detection/quantification, RT adjustment, peak alignment and weak signal recovery. The nonlinear curves for RT adjustment is recorded for each sample.

In the second stage (Fig. 1a, step 2), we generate a batch-level feature matrix for each batch. It is in the same format as the feature matrix from a single sample. For each feature detected in the batch, we keep the m/z value, and take the average RT value in the batch, and the average intensity value in the batch. Then across all the batch-level feature matrices, we conduct another round of RT adjustment and feature alignment (Fig. 1a, step 3). As each batch-level feature matrix is in the same structure as a single sample feature matrix, the RT adjustment and feature alignment can be easily achieved by calling the existing routines. At this stage, tolerance levels different than stage 1 can be used. Then the aligned batch-level features are mapped back to the original within-batch features, and weak signal recovery can be conducted across batches.

There are some challenges in this process. The major challenge is the second-round RT adjustment is conducted on the average RT values from each batch. We need to trace the adjustment back to each single sample in order to conduct weak signal recovery, which we address in the next subsection. The second and smaller challenge is the feature alignments across batch might result in the merging of two features from a batch, in which case we trace back to the feature matrix of the corresponding batch, merge the signal intensities of the corresponding features, and take the mean RT of the corresponding features.

### The RT correction procedure

In the regular preprocessing procedure, RT adjustment is conducted once by nonlinear curve fitting^5,11^. However in the two-stage procedure, there are two levels of RT deviation to be considered. One is within batch, and the other is between batch. In our new procedure, for each LC/MS profile, both levels of RT deviations are computed and added together, to create an overall RT correction at the profile level (Fig. 1b).

First within batch (Fig. 1a, step 1), the sample with the largest number of detected features is selected as the reference. The peak RTs of other samples are adjusted based on this reference sample. For each of the other samples, first a unique match between peaks in the sample and peaks in the reference sample is established based on certain m/z and RT tolerance levels. In the current study, to simplify the comparison between the two-stage and traditional apLCMS, we specified the same tolerance levels for them. Then a nonlinear curve is fitted between the RT difference and the observed RT in the sample to be corrected.

Within the *k*th batch, for the *j*th sample to be corrected, we denote the RTs of the uniquely matched peaks as $\left\{ {t_{m}^{{\left( {k,j} \right)}} } \right\}_{m = 1, \ldots ,M}$, and the RT of the corresponding peaks in the reference sample as $\left\{ {t_{m}^{{\left( {k,0} \right)}} } \right\}_{m = 1, \ldots ,M}$. We obtain a nonlinear curve fit for the deviation, represented by function *f*(),

$$ \Delta t^{{\left( {k,j} \right)}} = t^{{\left( {k,j} \right)}} - t^{{\left( {k,0} \right)}} = f_{k,j} \left( {t^{{\left( {k,j} \right)}} } \right) + \varepsilon $$

using kernel smoothing, and correct the RT of all the peaks in the *j*th sample to $\left\{ {t_{m}^{{\left( {k,j} \right)}} - \hat{f}_{k,j} \left( {t_{m}^{{\left( {k,j} \right)}} } \right)} \right\}_{m = 1, \ldots ,N}$, where *N* is the number of all the peaks in sample *j*.

After processing each batch, we obtain a batch-level feature table for each batch (Fig. 1a, step 2). In the feature table is the average RT value for each of the features in the batch. Between batches, we conduct a similar curve fit using the average feature RTs within each batch, against a reference batch (Fig. 1a, step 3). The batch with the largest number of aligned features is taken as the reference batch. For the *k*th batch, we denote the average RTs of the uniquely matched features as $\left\{ {\tau_{n}^{\left( k \right)} } \right\}_{n = 1, \ldots ,P}$, and the average RTs of the corresponding features in the reference batch $\left\{ {\tau_{n}^{\left( 0 \right)} } \right\}_{n = 1, \ldots ,P}$. We obtain a nonlinear curve fit for the deviation, represented by function *g(),*

$$ \Delta \tau^{\left( k \right)} = \tau^{\left( k \right)} - \tau^{\left( 0 \right)} = g_{k} \left( {\tau^{\left( k \right)} } \right) + \varepsilon $$

using kernel smoothing. Some example between-batch RT correction curves from real data (the CHDWB data described later) are shown in Fig. 1c. In the batch-level feature table, the RT is then corrected to $\left\{ {\tau_{n}^{\left( k \right)} - \hat{g}_{k} \left( {\tau_{n}^{\left( k \right)} } \right)} \right\}_{n = 1, \ldots ,N}$. Feature alignment are then conducted using the corrected batch-level RT, and then mapped back to the within-batch feature tables. As all batches share the same RT range, the parameter setting for the kernel smoother is the same for within-batch and cross-batch curve fitting.

### Weak signal recovery procedure

Some features pass the detection threshold in only a subset of the batches. For such features, cross-batch weak signal recovery is needed after alignment. However, in the final data table, the RT is corrected across all batches. We need to adjust the RT points in the original data in order for the weak signal recovery to be reliable. Hence an RT correction is conducted for every LC/MS profile in every batch (Fig. 1a, step 3). For the *j*th profile in the *k*th batch, the corrected RT is obtained by:

$$ t_{m, corrected}^{{\left( {k,j} \right)}} = t_{m}^{{\left( {k,j} \right)}} - \hat{f}_{k,j} \left( {t_{m}^{{\left( {k,j} \right)}} } \right) - \hat{g}_{k} \left( {t_{m}^{{\left( {k,j} \right)}} - \hat{f}_{k,j} \left( {t_{m}^{{\left( {k,j} \right)}} } \right)} \right), $$

where *m* indexes the RT points (Fig. 1b). After changing the RT, the weak signal recovery can be conducted as previously described^11^. Briefly, to recover the weak signal for a target m/z and RT pair in an LC/MS profile, a loose tolerance level in m/z and RT is first used to select a local region. Then two-dimensional kernel smoothing is conducted in the region to detect weak peaks. If a weak peak is close enough to the target m/z and RT pair (threshold determined by the peak detection tolerance levels), and the local point density passes a threshold, it is considered the recovered signal of the feature. More details can be found in^11^.

### Datasets

We use three datasets for methods comparison. The first was a standard sample (QSTD) constructed from pooled human plasma which was run repeatedly with different batches of samples for quality control purposes. In this analysis, we took the QSTD sample profiles from 10 batches, each containing 10 runs of the same sample. The data were generated using a C18 column combined with the Thermo Fisher Q Exactive Orbitrap Mass Spectrometer, in negative ion mode.

The second dataset was the ST000868 dataset^34^, downloaded from Metabolomics Workbench^35^. The study compared the metabolomic profile of oak and wine yeast strains. The data were collected in three batches. Each yeast strain was measured 3–6 times in every batch.

The third dataset was a subset of the untargeted metabolomics data from Emory/Georgia Tech Center for Health Discovery and Well Being (CHDWB). The CHDWB metabolomics data was collected on healthy individuals that received preventive care, and the metabolomics data can be requested by submitting a request form to the CHDWB (https://predictivehealth.emory.edu/research/resources.html)^36^. The study is a prospective longitudinal cohort study. Biological specimen, including blood samples, are collected every year for each participant. Metabolomics was measured on all subjects at baseline. We focused on the baseline metabolomics data and its relation with baseline body mass index (BMI) in this analysis. There were a total of 25 batches in the entire dataset. Within each batch, roughly 20 subjects were measured. The plasma sample from each subject was measured 3 times consecutively. We refer to them as triplets in the following text. The data were generated using a HILIC column combined with the Thermo Fisher Q Exactive Orbitrap Mass Spectrometer, in positive ion mode.

### Packages and parameters

We used apLCMS version 6.6.8 and xcms version 3.10.1, in the environment of R version 4.0.0. The apLCMS package and tutorial is available through https://mypage.cuhk.edu.cn/academics/yutianwei/apLCMS/, and XCMS is downloaded from Bioconductor.

There are three main parameters for this new approach. For the initial detection of peaks in each batch (Fig. 1a, step 1), *p*_*within_detect*_ controls the proportion of profiles a feature needs to be detected from, for it to be considered for the next step; *p*_*within_report*_ controls the proportion of profiles a feature need to be present after weak signal recovery, for it to be included in the final feature table from the batch. Between the batches (Fig. 1a, step 3), *p*_*batches*_ controls the proportion of batches the feature needs to be present, for it to be included in the overall feature table.

For apLCMS, the peak detection and quantification procedure for single LC/MS profile follows the existing method^11,12^. In this study, the major parameters include min.run = 12, min.pres = 0.5, mz.tol = 1e-5, baseline.correct = 0, min.bw = NA, max.bw = NA, shape.model = "bi-Gaussian", sd.cut = c(0.125, 60), sigma.ratio.lim = c(0.2, 5), moment.power = 1. Other parameters are listed in the R codes in the Supplementary Material.

For XCMS, four combinations of peak detection and RT correction methods were used. The parameters were optimized by the method IPO in an objective and dataset-specific manner^37^. XCMS IPO_1 uses optimal parameters found by IPO combining matched filter and orbiwarp. XCMS IPO_2 uses optimal parameters found by IPO combining matched filter and loess smoothing. XCMS IPO_3 uses optimal parameters found by IPO combining centWave and orbiwarp. XCMS IPO_4 uses optimal parameters found by IPO combining centWave and loess smoothing. As the parameters are dataset-specific, their values are listed in the Results and Discussions section.

## Results and discussions

We implemented the method in the existing workflow of the apLCMS package^11^, which conducts both untargeted and hybrid (untargeted/targeted) feature detection^12^. To evaluate the feature detection performance of the proposed two-stage approach, we conducted comparison experiments with the traditional apLCMS approach, as well as the popular preprocessing method XCMS^19^, on three real datasets.

### Results from standard sample (QSTD) data

Using the QSTD data, we compared the performance of the new two-stage apLCMS with tradition apLCMS and XCMS in feature detection and quantification. For apLCMS, we first selected optimal parameter settings for peak detection and kept the parameters the same for both the two-stage and the traditional methods.

For the two-stage approach, we tested two scenarios for within-batch proportion parameters, *p*_*within_detect*_ = *p*_*within_report*_*, *and 2*p*_*within_detect*_ = *p*_*within_report*_ . We found the results to be similar with regard to the criteria we used to assess the performance. Thus in the following sections, we report results from using the same values for *p*_*within_detect*_ (before weak signal recovery) and *p*_*within_report*_ (after weak signal recovery). We used values of 0.2, 0.3, 0.4, 0.6, 0.8 and 1. The second parameter was between-batch detection proportion threshold *p*_*batches*_, i.e. the proportion of batches a feature needed to be present in. We used values of 0.1, 0.2, 0.3, 0.5, 0.7, and 0.9. For the traditional apLCMS procedure, the detection threshold (number of profiles the feature needed to be present in) was set as 5, 10, 15, …., and 95.

For XCMS, we used the IPO package to optimize its parameters under 4 different method combinations. Below are the parameter combinations in each of the 4 settings:

XCMS IPO_1: matched filter parameters: fwhm = 15, snthresh = 1, step = 0.0805, steps = 2, sigma = 6.369, max = 5, mzdiff = 0.639, index = FALSE; peak grouping parameters: method = "density", bw = 0.879999, mzwid = 0.0614; Orbiwarp parameters: method = "obiwarp", plottype = "none", distFunc = "cor_opt", profStep = 1, center = 6, response = 1, gapInit = 0.78, gapExtend = 2.7, factorDiag = 2, factorGap = 1, localAlignment = 0.

XCMS IPO_2: matched filter parameters: same as XCMS IPO_1; peak grouping parameters: method = "density", bw = 0.879999, mzwid = 0.0362; Loess parameters: missing = 3, extra = 3, span = 0.221, smooth = "loess", family = "gaussian".

XCMS IPO_3: centWave parameters: peakwidth = c(3, 129.97), ppm = 10, noise = 0, snthresh = 1, mzdiff = -0.0109, prefilter = c(3,100), mzCenterFun = "wMean", integrate = 1, fitgauss = FALSE, verbose.columns = FALSE; peak grouping parameters: method = "density", bw = 12.4, mzwid = 0.01; Orbiwarp parameters: distFunc = "cor_opt", profStep = 1, center = 7, response = 1, gapInit = 0.54, gapExtend = 2.7, factorDiag = 2, factorGap = 1, localAlignment = 0.

XCMS IPO_4: centWave parameters: same as XCMS IPO_3; peak grouping parameters: bw = 0.25, mzwid = 0.0081; Loess parameters: missing = 5, extra = 1, span = 0.326, smooth = "loess", family = "gaussian".

To achieve different number of features detected by XCMS, while keeping the above parameters fixed, we varied the “minsamp” parameter, which controls the minimum number of samples necessary for a peak group to be detected. We used values of 5, 10, 20, 30, 40, 50, 60, 70, 80, 90.

**Figure 2:**
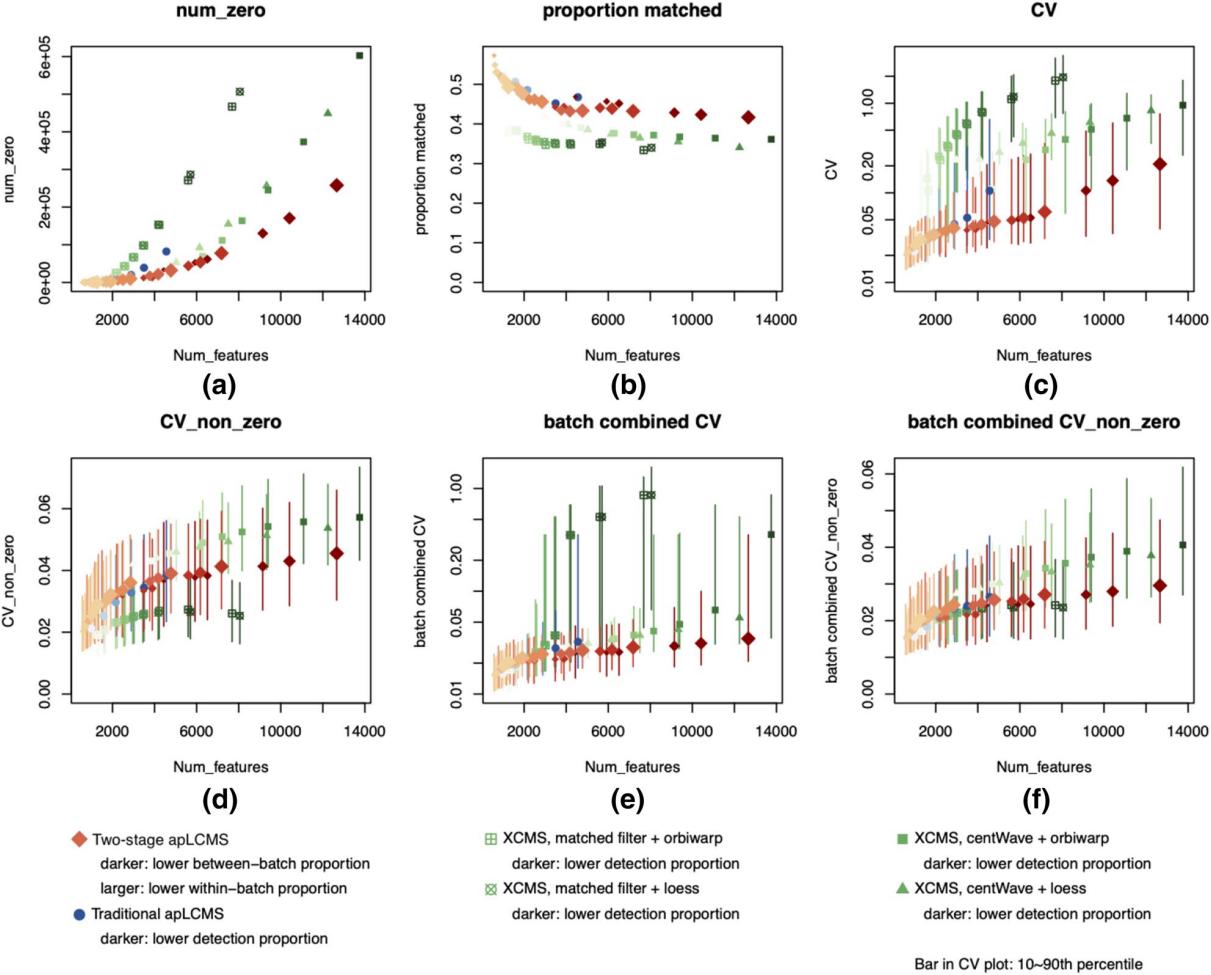
Comparison of the two-stage preprocessing approach with traditional apLCMS and XCMS using standard sample. Each dot represents a parameter setting. (**a**) Total number of zeros in the final data matrix; (**b**) proportion of features with m/z matched to known metabolites using xMSAnnotator; (**c**) level of variation as measured by coefficient of variation (CV) in the final data matrix without considering batches; (d) level of variation as measured by coefficient of variation (CV) in the final data matrix without considering batches, considering only non-zero values; (e) level of variation as measured by CV after merging each batch; (f) level of variation as measured by CV after merging each batch, considering only non-zero values. In all CV plots, the point is median; vertical bars represent 10th to 90th percentile.

To evaluate the results, we recorded the total number of zeros in the final data matrix (Fig. 2a), number of features with m/z matched to known KEGG metabolites using xMSAnnotator^18^ allowing adduct ions [M–H]^−^, [M–2H]^2−^, [M–2H + Na]^−^, [M–2H + K]^−^, [M–2H + NH4]^−^, [M–H_2_O–H]^−^, [M–H + Cl]^2−^, [M + Cl]^−^, [M + 2Cl]^2−^ (Fig. 2b), coefficient of variation (CV) in the final data matrix without considering batches with and without considering the zero values (Fig. 2c,d), and the coefficient of variation (CV) after merging the repeated measurements in each batch to generate a single measurement from each batch, with and without considering the zero values (Fig. 2e,f). In the calculation of CV, including zero values can reflect feature detection consistency in the CV results, while excluding zero values can reflect feature quantification consistency.

In untargeted metabolomics data measured by LC/MS, zeros in the final data matrix represent a mixture of true non-presence of the metabolic feature and missing values. It is still a difficult issue to address. Given the measurements here were taken on the same sample, we expect a better method to yield less zeros in the data matrix. However, the proportions of zero also depends on how consistent the LC/MS machinery generates the data, and how aggressive the weak signal recovery is conducted. Thus the results need to be considered together with the level of variation in the CV plots. When weak signal recovery is conducted in an overly aggressive manner taking noise ask signal, although the proportion of zeros may be lower, the inclusion of noise as signal will also worsen the quantification consistency. As shown in Fig. 2a, when the number of features were large, the two-stage approach (orange) tended to yield smaller proportions of zeros compared to the traditional apLCMS approach (blue) and XCMS (green).

The proportion of features that could be matched were similar for the three methods (Fig. 2b). Traditional apLCMS was slightly better, and XCMS was slightly inferior. When the detection threshold was loosened, some noise data points were expected to be mis-identified as features. At the same time, some low-abundance metabolites were detected. Thus we expected a higher false-positive rate in the metabolite mapping, which was a trade-off with a higher detection rate over all metabolites in the sample.

In the measurement of the coefficient of variation (CV) before and after merging within batches, as illustrated in Fig. 2c–f, the two-stage method (orange diamonds) yielded less variation compared to the traditional apLCMS (blue dots) and XCMS (green triangles) when zero was included in the calculation of CV (Fig. 2c,e). When zero values were excluded, XCMS with matched filter approach yielded better quantification consistency as evidenced by lower CV values (Fig. 2d). The advantage disappeared when the data from each batch was merged (Fig. 2f). However with regard to detection consistency, XCMS with matched filter resulted in much higher proportion of zeros (Fig. 2a). Given the data was collected on the same sample, we expect a feature’s presence should vary little across the files. Overall, the two-stage approach outperformed the traditional apLCMS and XCMS in terms of measurement stability.

### Results from ST000868 dataset

For apLCMS, we used *p*_*within_detect*_ = *p*_*within_report*_ = *0.1, 0.2, 0.3, 0.4, 0.5, 0.6, 0.7, 0.8, 0.9, and p*_*batches*_ = *0.1, 0.2, 0.3, 0.4, 0.5, 0.6, 0.7, 0.8, 0.9.* All other parameter setting were the same as the previous section except min.run = 0.8 and min.pres = 0.4, given the shorter RT range of the data. We note some of the above parameter combinations may result in identical results given the small batch size. For traditional apLCMS, while keeping all other parameters the same as the two-stage approach, we used the detection threshold (number of profiles the feature needed to be present in) of 2, 4, 6, …, 28.

For XCMS, we again used the IPO package to optimize its parameters under 4 different method combinations. Below are the parameter combinations in each of the 4 settings:

XCMS IPO_1: Matched Filter parameters: fwhm = 25, snthresh = 3, step = 0.05, steps = 1, sigma = 10.617, max = 5, mzdiff = 0.75, index = FALSE; peak grouping parameters: method = "density", bw = 38, mzwid = 0.015; Orbiwarp parameters: method = "obiwarp", plottype = "none", distFunc = "cor_opt", profStep = 1, center = 3, response = 1, gapInit = 0, gapExtend = 2.7, factorDiag = 2, factorGap = 1, localAlignment = 0.

XCMS IPO_2: matched Filter parameters: same as XCMS IPO_1; peak grouping parameters: method = "density", bw = 12.4, mzwid = 0.027; Loess parameters: missing = 3, extra = 3, span = 0.22, smooth = "loess", family = "gaussian".

XCMS IPO_3: CentWave parameters: peakwidth = c(10, 50), ppm = 5, noise = 0, snthresh = 1, mzdiff = -0.01, prefilter = c(1, 100), mzCenterFun = "wMean", integrate = 1, fitgauss = FALSE, verbose.columns = FALSE; peak grouping parameters: method = "density", bw = 37.68, mzwid = 0.0001; Orbiwarp parameters: distFunc = "cor_opt", profStep = 1, center = 3, response = 1, gapInit = 0, gapExtend = 2.7, factorDiag = 2, factorGap = 1, localAlignment = 0.

XCMS IPO_4: CentWave parameters: same as XCMS IPO_3; peak grouping parameters: bw = 12.4, mzwid = 0.0001; Loess parameters: missing = 1, extra = 2, span = 0.42, smooth = "loess", family = "gaussian".

To achieve different number of features detected by XCMS, while keeping the above parameters fixed, we varied the “minsamp” parameter, which controls the minimum number of samples necessary for a peak group to be detected. We used values of of 2, 4, 6, …, 28.

To compare the results from the three methods, we compared detection/quantification consistency, matching to known metabolites, and testing results by contrasting the two cell types, as the original study was designed to find the metabolic differences between the genetically different cell types.

**Figure 3:**
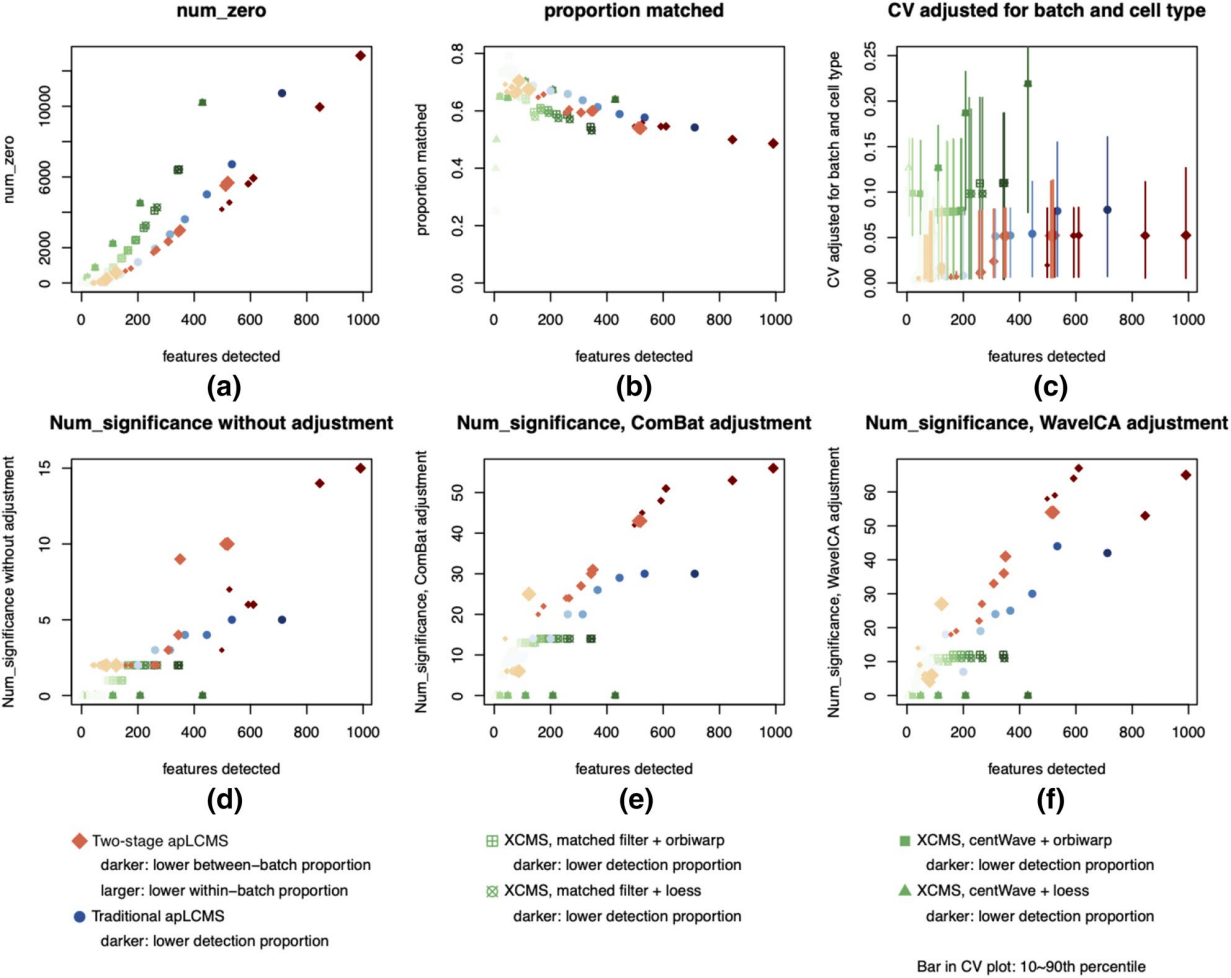
Comparison of the two-stage preprocessing approach with traditional apLCMS and XCMS using the ST000868 dataset. Each dot represents a parameter setting. (**a**) Proportion of zeros in the final data matrix before merging triplets for each subject; (**b**) Proportion of features with m/z matched to known metabolites by xMSAnnotator; (**c**) Within-triplet coefficient of variation (CV). Point is median; vertical bars represent 10th to 90th percentile. (**d**) Number of significant features at FDR ≤ 0.2, without batch effect correction; (**e**) Number of significant features at FDR ≤ 0.2, after batch effect correction by ComBat; (**f**) Number of significant features at FDR ≤ 0.2, after batch effect correction by WaveICA.

Similar to the QSTD data, the two-stage method resulted in smaller proportion of zeros (Fig. 3a). In the m/z matching to KEGG metabolites using adduct ions [M–H]^−^, [M–2H]^2−^, [M–2H + Na]^−^, [M–2H + K]^−^, [M–2H + NH4]^−^, [M–H_2_O–H]^−^, [M–H + Cl]^2−^, [M + Cl]^−^, [M + 2Cl]^2−^, the methods performed similarly, with XCMS with centWave peak detection yielding slightly higher rate of matching (Fig. 3b). With regard to CV values after adjusting for cell type and batch, i.e. the variation for each cell type within each batch, the two-stage approach resulted in lower CVs (Fig. 3c), indicating better detection and quantification consistency.

We then conducted testing between the two cell types using t-test. All tests were first conducted at the single metabolic feature level, and then the *p* values from all features were subjected to False Discovery Rate (FDR) correction^38^. The tests were limited to features with ≤ 33% zeros in at least one of the cell types. Without batch effect correction, all method yielded relatively few significant metabolites at FDR ≤ 0.2, while the two-stage method tended to detect more significant feature (Fig. 3d). We then applied two batch effect correction methods. The first was the popular method ComBat^28^, which was originally developed for microarray data, and was later widely used in RNA-seq and metabolomics data. After applying ComBat to each of the data matrices, testing was conducted on the adjusted data. All methods detected more significant metabolic features after the adjustment (Fig. 3e). The two-stage approach, when combined with ComBat, resulted in more significant metabolic features than the other two methods (Fig. 3e). Among the four combinations of XCMS, matched filter appeared to result in more significant metabolic features. We notice that the number of significant metabolic features from XCMS-processed data tended to fall close to a horizontal line. This is due to the fact that in XCMS, features detected using a more restrictive minsamp setting are a strict subset of those detected using a looser minsamp setting, when other parameters stay the same. When the threshold ≤ 33% zeros in at least one cell type was applied to the data matrix, some matrices obtained with different minsamp settings yielded similar matrices after filtration.

We applied another recent batch effect correction method that was specifically developed for metabolomics data – WaveICA, which has shown excellent performance when compared to some other existing methods^29^. After applying WaveICA to all the data matrices, the results were similar to ComBat. Again the two-stage approach detected more significant features (Fig. 3f). Overall, when applied to the ST000868 dataset, the new two-stage approach resulted in more consistent peak detection and better between-cell type testing results.

### Results from the CHDWB data

In this study, we selected six batches from the CHDWB data that evenly spanned the entire dataset: batches 1, 5, 10, 15, 20, 25, which included 115 subjects in total. Between the traditional apLCMS and the new two-stage approach, we kept all other parameters the same, except the detection proportion threshold values. In the two-stage procedure, we applied within-batch detection proportion threshold values 0.2, 0.3, 0.4, 0.6, 0.8, and 1, and between-batch detection proportions 0.15, 0.3, 0.45, 0.6, 0.75, and 0.9. Given there were six batches, the between-batch detection proportions meant we required a feature to be initially detected in at least 1, 2, 3, 4, 5, or 6 batches, respectively. For the traditional apLCMS procedure, we set the detection threshold (number of samples) at 30, 60, 90, 120, 180, 240, and 300. For XCMS, we used the IPO package to optimize its parameters under 4 different method combinations. Below are the parameter combinations in each of the 4 settings:

XCMS IPO_1: Matched Filter parameters: fwhm = 27, snthresh = 1, step = 0.015, steps = 2, sigma = 11.4659, max = 5, mzdiff = 0.77, index = FALSE; peak grouping parameters: method = "density", bw = 0.879999, mzwid = 0.0265; Orbiwarp parameters: method = "obiwarp", plottype = "none", distFunc = "cor_opt", profStep = 1, center = 5, response = 1, gapInit = 0.928, gapExtend = 2.7, factorDiag = 2, factorGap = 1, localAlignment = 0.

XCMS IPO_2: Matched Filter parameters: same as XCMS IPO_1; peak grouping parameters: method = "density", bw = 0.879999, mzwid = 0.0265; Loess parameters: missing = 4, extra = 1, span = 0.05575, smooth = "loess", family = "gaussian".

XCMS IPO_3: CentWave parameters: peakwidth = c(3,110), ppm = 10, noise = 0, snthresh = 1, mzdiff = -0.0175, prefilter = c(3,100), mzCenterFun = "wMean", integrate = 1, fitgauss = FALSE, verbose.columns = FALSE; peak grouping parameters: method = "density", bw = 12.4, mzwid = 0.003; Orbiwarp parameters: distFunc = "cor_opt", profStep = 1, center = 2, response = 1, gapInit = 0.08, gapExtend = 2.7, factorDiag = 2, factorGap = 1, localAlignment = 0.

XCMS IPO_4: CentWave parameters: same as XCMS IPO_3; peak grouping parameters: bw = 22, mzwid = 0.018; Loess parameters: missing = 1, extra = 3, span = 0.2, smooth = "loess", family = "gaussian".

To achieve different number of features detected by XCMS, we varied the “minsamp” parameter, which controls the minimum number of samples necessary for a peak group to be detected. We used values 10, 20, 30, 50, 70, 90, 120, 180, 240, 300.

**Figure 4:**
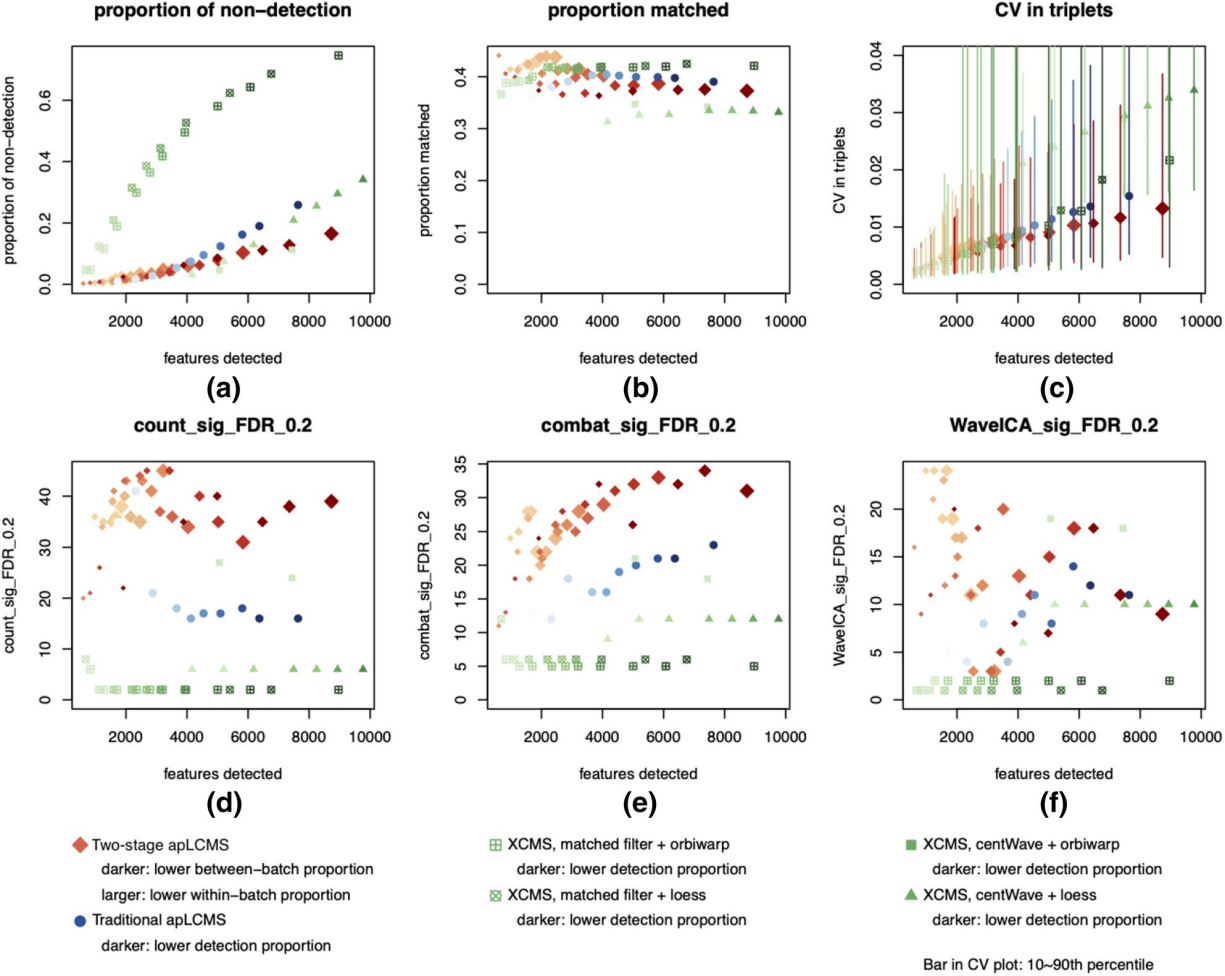
Comparison of the two-stage approach with traditional apLCMS and XCMS using CHDWB samples. Each dot represents a parameter setting. (**a**) Proportion of zeros in the final data matrix before merging triplets for each subject; (**b**) proportion of features with m/z matched to known metabolites by xMSAnnotator; (**c**) average within-triplet coefficient of variation (CV). Point is median; vertical bars represent 10th–90th percentile. (**d**) Number of significant features at FDR ≤ 0.2, without batch effect correction; (**e**) Number of significant features at FDR ≤ 0.2, after batch effect correction by ComBat; (**f**) Number of significant features at FDR ≤ 0.2, after batch effect correction by WaveICA.

Some settings resulted in data matrices with more than 10,000 features, which is out of the range a regular untargeted analysis would consider. Thus we limited the following discussion to data matrices with 10,000 features or less. We assessed the results based on following criteria for consistency: Total number of zeros in the final data matrix (Fig. 4a), features with m/z matched to known metabolites with KEGG IDs using xMSAnnotator, allowing adduct ions [M + H]^+^, [M + NH4]^+^, [M + Na]^+^, [M + ACN + H]^+^, [M + ACN + Na]^+^, [M + 2Na–H]^+^, and [M + K]^+^ (Fig. 4b), and coefficient of variation within the triplet that measured the same sample (Fig. 4c). As shown in Fig. 4a, when the total number of features was below 4,000, the two-stage approach and traditional apLCMS yielded smaller proportion of zeros. When the total number of features went larger, the XCMS with centWave and orbiwap combination and the two-stage approach yielded data matrices that tended to have smaller proportions of zeros. Although the data were generated from different subjects, we still expected the core metabolism to be similar across the subjects, and a better method would conduct more consistent feature alignment between samples/batches, resulting in less zeros in the final data matrix. This should be true especially when smaller number of metabolic features are detected, which are more concentrated in core metabolism.

With regard to features matched to known metabolites, the three methods performed similarly, with the two-stage approach having a slight edge when the number of features detected were smaller, and XCMS with matched filter having slightly more matched features when the number of features went larger (Fig. 4b). We computed the coefficient of variation (CV) over all the metabolic features within each triplet (subject). As shown in Fig. 4c, the median CV level tended to be similar for all the approaches when the number of features were smaller (< 4,000), while the two-stage approach had an edge when the number of features were larger. In addition, the distribution of CV tended to be wider for XCMS, indicating part of the metabolic features showed larger variation within triplets.

Next we merged the triplet measures for each subject. The merging was done by taking the average non-zero values in the triplet for each feature. When all three measurements for a feature were zero, the resulting merged measurement was also zero. Using each of the feature table, we first filtered the features using a threshold of < 25% zeros, and then conducted down-stream analysis using the body mass index (BMI) as the outcome variable, while adjusting for age, gender and race. It is well known that BMI is associated with changes in metabolic patterns^39^. We fitted a linear model for each individual metabolic feature (denoted M):

$$ BMI = \mu + \beta_{1,i} M_{i} + \beta_{2} Age + \beta_{3} Age^{2} + \beta_{4} Gender + \beta_{5} Race + \varepsilon $$

Here the subscript *i* indexes the metabolic feature. The *p* value associated with *β*_1,*i*_ was recorded. Then the *p* values from all features were subjected to False Discovery Rate (FDR) correction^38^.

Without batch effect correction, the two-stage approach yielded higher number of significant features over the entire range of number of features detected (Fig. 4d). We then applied ComBat^28^ to adjust for batch effect in each data matrix before applying the above testing procedure. After the application of ComBat, the two-stage approach showed a trend of increasing number of significant features with the increase of total number of features in the matrix (Fig. 4e). It was again the method that detected the highest number of significant features across the range of total number of features. Applying the batch effect correction method WaveICA, the results were more mixed. When the number of features were low to moderate (< 5,000), the two-stage approach detected more significant features. When considering larger number of features, two settings of XCMS with centWave+ orbiwarp resulted in higher number of significant features. Overall, the new two-stage approach again resulted in more consistent peak detection and quantification, as well as better down-stream testing result.

Next we considered the biological interpretability of the testing results. For this purpose, we conducted pathway analyses using Mummichog^40^. As Mummichog needed to be conducted manually, we selected a subset of the results for this analysis. We selected four groups of data matrices with ~ 5,000, ~ 4,000, ~ 3,000, and ~ 2000 features, respectively. Because pathway analysis requires a reasonable number of significant features, instead of using FDR, we used features with raw *p* value < 0.05.

As shown in Table 1, in the two groups with lower feature counts (~ 2000 and ~ 3,000), the two-stage approach yielded more significant pathways with at least 5 significant metabolic features (Table 1, last column). In the group of ~ 4,000 features, the two-stage approach tied with traditional apLCMS at 8 significant pathways. In the group with ~ 5,000 features, traditional apLCMS had a slight edge over the two-stage approach. XCMS with centWave+ loess resulted in 5 significant pathways, which was only slightly worse.

**Table 1:** Comparison of feature selection and pathway analysis results.

Method	Total # features	# Significant pathways with 5 or more matched significant metabolites
Two-stage, P_within.detect_ = 0.3 p_batches_ = 0.3	5,024	6
Two-stage, P_within.detect_ = 0.6 p_batches_ = 0.15	4,988	5
Traditional apLCMS, min.profiles = 50	5,097	***7***
XCMS matched filter + orbiwarp, minsamp 30	5,004	0
XCMS centWave + orbiwarp, minsamp 300	5,064	5
XCMS centWave + loess, minsamp 240	5,201	1
Two-stage, p_within.detect_ = 0.2 p_batches_ = 0.45	4,034	***8***
Traditional apLCMS, min.profiles = 90	4,129	***8***
XCMS centWave + loess, minsamp 300	4,165	2
XCMS matched filter + orbiwarp, minsamp 50	3,928	0
Two-stage, p_within.detect_ = 0.3 p_batches_ = 0.6	2,837	***5***
Traditional apLCMS, Min.profiles = 180	2,874	3
XCMS matched filter + orbiwarp, minsamp 90	2,789	0
Two-stage, p_within.detect_ = 0.3 p_batches_ = 0.9	1667	***5***
Traditional apLCMS, Min.profiles = 300	1725	3
XCMS matched filter + orbiwarp, minsamp 180	1704	0

BMI was used as the outcome variable. Age, age^2^, gender, and race were adjusted for in the model. Metabolic feature selection was conducted using features with < 25% zeros. Pathway analysis was conducted using Mummichog, using metabolic features with *p* < 0.05.

The bold italic font represents the biggest number of significant pathways in the comparison group

Given the settings with ~ 4,000 features yielded the most significant pathways, we further examined the selected pathways by the three methods in this group (Table 2). The two-stage approach and traditional apLCMS each yielded 8 significant pathways with at least 5 matched significant metabolites. Their results largely agreed with each other. The significant pathways tended to be focused on amino acid metabolism, which was expected to be highly relevant to BMI status. The top pathway selected by the two-stage approach also included “Phosphatidylinositol phosphate metabolism”, which is known to be involved in the activation of various pathways. Dysregulation of the metabolism of phosphatidylinositol-3,4,5-triphosphate mediates insulin resistance^41^, which is highly relevant to BMI. The XCMS yielded much fewer significant pathways. The urea cycle pathway was shared with the other two approaches.

**Table 2:** Significant pathways with at least 5 matched significant metabolic features for parameter settings where ~ 4,000 features were detected.

Pathways	Overlap_size	Pathway_size	*p* value
Two-stage apLCMS (within-batch proportion 0.3, initially detected in at least 4 batches), 4,034 features			
Lysine metabolism	6	19	0.00185
Phosphatidylinositol phosphate metabolism	5	16	0.00479
Butanoate metabolism	5	17	0.00681
Glycine, serine, alanine and threonine metabolism	8	38	0.00798
Aspartate and asparagine metabolism	9	52	0.01899
Urea cycle/amino group metabolism	7	40	0.02756
Pyrimidine metabolism	5	27	0.0463
Glycerophospholipid metabolism	8	53	0.04966
Traditional apLCMS (minimum samples detected 90), 4,129 features			
Butanoate metabolism	5	15	0.00387
Glycine, serine, alanine and threonine metabolism	8	37	0.00689
Arachidonic acid metabolism	6	24	0.00748
Lysine metabolism	5	18	0.0079
Vitamin B3 (nicotinate and nicotinamide) metabolism	5	18	0.0079
Glycerophospholipid metabolism	9	52	0.01681
Urea cycle/amino group metabolism	7	43	0.041
Aspartate and asparagine metabolism	8	53	0.04899
XCMS (centWave + loess, IPO optimized, minimum samples detected 90), 4,165 features			
C21-steroid hormone biosynthesis and metabolism	6	24	0.00395
Urea cycle/amino group metabolism	5	30	0.04353

Overall, with this larger dataset generated from real biological subjects, we again demonstrated that the two-stage approach generated data with higher consistency, as compared to the traditional apLCMS and XCMS that treated all the data as a single group.

### Discussions

The two-stage approach is built on top of the existing apLCMS method. It first conducts the entire workflow of within-batch feature detection, RT correction, and feature alignment. Then it conducts between-batch feature alignment, RT correction, and weak signal recovery across batches. The RT correction is conducted in a two-stage manner, by adding together two smooth curves for each LC/MS profile. One curve is within-batch RT deviation, and the other curve is between-batch RT deviation.

The method has a few important parameters. The tuning of the parameters is somewhat heuristic. The situation is similar to the tuning of other parameters in the apLCMS, XCMS, or packages. Different studies may have different purposes. Some studies focus more on the core metabolic network, while others aim at identifying low-abundance metabolites and environmental chemicals. Hence there isn’t a globally optimal choice of the parameters. However, the newly added parameters for two-stage processing have straight-forward interpretations. They are proportions of samples from which the features are detected, either in each batch, or across the batches. The higher the value of *p*_*within_detect*_, the more stringent the within-batch peak detection, the less features detected within each batch. Similarly, *p*_*within_report*_ tunes the stringency after within-batch weak signal recovery. A higher *p*_*within_report*_ value results in less features reported from each batch. The parameter *p*_*batch*_ controls between-batch stringency. A higher *p*_*batch*_ value requires an aligned feature to be detected in more batches. Thus increasing the value of *p*_*batch*_ results in lower number of features. Given their interpretability, the tuning would be a guided effort by the user.

By combining the two-stage method with batch-effect correction methods ComBat and WaveICA, we found that at least in some datasets, the application of batch-effect correction can further improve the data quality. After the application of the batch-effect correction methods, the two-stage approach still outperformed traditional apLCMS and XCMS. This indicates that addressing batch effect in data preprocessing is important.

Given the total number of samples, the computing time is influenced by the batch size. We examined the computing time using the 100 QC profiles, using an old HP workstation with dual first-generation Xeon E5-2660 CPU. We utilized 10 CPU cores. The computing time was ~ 70 min.

Besides de novo feature detection, a hybrid feature detection method is available in apLCMS, in which a pre-existing database of known feature is used to improve weak signal detection^12^. In the current study, for fairness of comparison, we did not use known feature database. Nonetheless, besides conducting untargeted feature detection, the new two-stage procedure is also adapted to the hybrid feature detection procedure. It is capable of incorporating prior knowledge to boost feature detection.

There are some limitations to the method. The current implementation is limited to apLCMS, and thus limited to high-resolution LC/MS data. We believe the same strategy can be implemented in other packages for wider application, such as GC/MS data. This work was focused on data generated in multiple batches from the same machine. In the CHDWB dataset, we picked batches that were not consecutively collected, and the method worked well. Nonetheless, although there can be some batch effects, we still assume different batches cannot have drastically different characteristics, as reliable feature alignment is necessary for batch effect correction. The issue of combining data from multiple machines is a much more difficult one. We will try to address such issues in future studies.

## Conclusion

We presented a two-stage approach for LC/MS metabolomics data generated in multiple batches. By analyzing data with multiple batches, both generated from a standardized plasma sample and from real biological samples, we showed that the new method improved the consistency of feature detection and quantification. The method is available as part of the apLCMS package. The package can be downloaded at https://github.com/tianwei-yu/apLCMS. The instructions are at https://mypage.cuhk.edu.cn/academics/yutianwei/apLCMS/.

## Supplementary information

Supplementary information
